# Prognosis of critically ill cirrhotic versus non-cirrhotic patients: a comprehensive score-matched study

**DOI:** 10.1186/1471-2253-14-123

**Published:** 2014-12-17

**Authors:** Chung-Ming Fu, Chih-Hsiang Chang, Pei-Chun Fan, Ming-Hung Tsai, Shu-Min Lin, Kuo-Chin Kao, Ya-Chung Tian, Cheng-Chieh Hung, Ji-Tseng Fang, Chih-Wei Yang, Yung-Chang Chen

**Affiliations:** Kidney Research Center, Department of Nephrology, Chang Gung Memorial Hospital, Taipei, Taiwan; Division of Gastroenterology, Chang Gung Memorial Hospital, Taipei, Taiwan China; Department of Thoracic Medicine, Chang Gung Memorial Hospital, Taipei, Taiwan China; Chang Gung University College of Medicine, Taoyuan, Taiwan

**Keywords:** Acute physiology and chronic health evaluation III (APACHE III), Sequential organ failure assessment (SOFA), Intensive care unit (ICU), Cirrhosis, Outcome

## Abstract

**Background:**

Cirrhotic patients admitted to an intensive care unit (ICU) have high mortality rates. The present study compared the characteristics and outcomes of critically ill patients admitted to the ICU with and without cirrhosis using the matched Acute Physiology and Chronic Health Evaluation III (APACHE III) and Sequential Organ Failure Assessment (SOFA) scores.

**Methods:**

A retrospective case-control study was performed at the medical ICU of a tertiary-care hospital between January 2006 and December 2009. Patients were admitted with life-threatening complications and were matched for APACHE III and SOFA scores. Of 336 patients enrolled in the study, 87 in the cirrhosis or noncirrhosis group were matched according to the APACHE III scores. Another 55 patients with cirrhosis were matched to the 55 patients without cirrhosis according to the SOFA scores. Demographic data, aetiology of ICU admission, and laboratory variables were also evaluated.

**Results:**

The overall hospital mortality rate in the patients with cirrhosis in the APACHE III-matched group was more than that in their counterparts (73.6% vs 57.5%, *P* = .026) but the rate did not differ significantly in the SOFA-matched group (61.8% vs 67.3%). In the APACHE III-matched group, the SOFA scores of patients with cirrhosis were significantly higher than those of patients without cirrhosis (*P* < .001), whereas the difference in APACHE III scores was nonsignificant between the SOFA-matched patients with and without cirrhosis.

**Conclusions:**

Score-matched analytical data showed that the SOFA scores significantly differentiated the patients admitted to the ICU with cirrhosis from those without cirrhosis in APACHE III-matched groups, whereas difference in the APACHE III scores between the patients with and without cirrhosis were nonsignificant in the SOFA-matched group.

## Background

Accurate prognostic predictors are crucial for patients admitted to an intensive care unit (ICU). Prognostic scoring systems are useful for clinical management such as predicting a survival rate, making decisions, and facilitating explanation of disease severity, by clinical physicians. Patients with cirrhosis admitted to an ICU frequently have disappointing outcomes despite intensive medical support, and these patients are particular targets for prognostic evaluation.

Various systems for scoring severity and predicting prognosis have been developed and applied for decades. The Acute Physiology and Chronic Health Evaluation III (APACHE III) score [1], one of the widely used scoring systems, is known for its accuracy in predicting mortality. However, the APACHE III scoring system was initially developed for various diseases and not exclusively for liver-related diseases. By contrast, the Sequential Organ Failure Assessment (SOFA) score [2], another widely used scoring system, is superior to the APACHE III scoring system for assessing specific organ dysfunction including cirrhosis [3, 4]. Our previous study demonstrated that the APACHE III and SOFA scores were both independently associated with a hospital mortality rate and demonstrated high discriminatory power for predicting mortality in patients with cirrhosis [5]. However, few studies have performed detailed independent comparisons between APACHE III and SOFA scores [6]. In the present case-control study, we matched APACHE III and SOFA scores and compared the different clinical characteristics and outcomes of patients with cirrhosis with those of their matched noncirrhotic controls.

## Methods

### Study design

The Chang Gung Medical Foundation Institutional Review Board approved the present study and waived the need for informed consent, because patient privacy was not breached during the study, and the study did not interfere with clinical decisions related to patient care (approval No. 98-3658A3). All data in our study were anonymised. This retrospective case-control study was conducted in a tertiary-care hospital. The enrolled patients were recruited from a database of critically ill patients admitted to medical ICUs between January 2006 and December 2009. For the APACHE III-matched group (174 patients), each patient with cirrhosis was matched 1:1 to a control patient without cirrhosis by using the criteria of APACHE III ± 3 points. For the SOFA-matched group (110 patients), each patient with cirrhosis was matched 1:1 to a control patient without cirrhosis by using the criteria of SOFA ± 1 point [7, 8]. The outcomes of interest were the length of stay in an ICU, length of stay in a hospital, and hospital mortality rate.

### Study population and data collection

All patients admitted to medical ICUs between January 2006 and December 2009 with APACHE III and SOFA scores available were eligible for inclusion. Exclusion criteria were age < 18 years, length of stay in a hospital or an ICU of < 24 hours, patients with chronic uraemia and undergoing renal replacement therapy, and hospital readmission. Data were recorded regarding patient demographics, reason for ICU admission, clinical and laboratory variables, APACHE III and SOFA scores, the risk of renal failure, injury to the kidney, failure of kidney function, loss of kidney function, end-stage renal failure (RIFLE) classification [9], the length of stay in an ICU and a hospital, and hospital mortality. Data on the length of stay included those from patients with hospital mortality.

### Definitions

Cirrhosis was diagnosed based on liver histology or a combination of physical presentation, biochemical data, and ultrasonographic findings. Illness severity was assessed according to the APACHE III and the SOFA scores, which were defined and calculated as described previously [1, 2]. Acute kidney injury (AKI) was defined using the RIFLE criteria, and patients were scored as RIFLE-R or higher severity. Baseline serum creatinine (SCr) concentration was the first value measured during hospitalisation. The Modification of Diet in Renal Disease formula was used to estimate baseline SCr concentration in patients whose previous SCr concentration was unavailable. The criteria resulting in the most severe RIFLE classification were used [9]. A simple model for assessing mortality was developed as follows: non-AKI (0 points), RIFLE-R (1 point), RIFLE-I (2 points), and RIFLE-F (3 points) on Day 1 of ICU admission [10, 11].

The lowest physiological and biochemical values on Day 1 of ICU admission were recorded. In sedated or paralysed patients, neurological scoring was not performed and was not classified as neurological failure. In patients who were intubated but not sedated, the best verbal response was determined according to clinical judgment.

### Statistical analysis

Descriptive statistical results were expressed as mean ± standard error (SE). In primary analysis, the patients with cirrhosis were compared with the patients without cirrhosis. All variables were tested for normal distribution using the Kolmogorov–Smirnov test. Student’s *t*-test was used to compare the means of continuous variables and normally distributed data, whereas the Mann–Whitney *U* test was used for all other comparisons. Categorical data were tested using the χ^2^ test or Fisher’s exact test.

Calibration was assessed using the Hosmer–Lemeshow goodness-of-fit test (C statistic) to compare the number of observed and predicted deaths in various risk groups for the entire range of death probabilities. Discrimination was assessed by determining area under the receiver operating characteristic curve (AUROC). Areas under 2 receiver operating characteristic curves were compared by applying a nonparametric approach. The AUROC analysis was also conducted to estimate the cut-off values, sensitivity, specificity, overall correctness, and positive and negative predictive values. Finally, cut-off points were calculated by determining the best Youden’s index (sensitivity + specificity −1).

Cumulative survival curves over time were generated by applying the Kaplan–Meier approach and compared using the log rank test. All statistical tests were 2-tailed; *P* < .05 was considered statistically significant. Data were analysed using SPSS Version 13.0 for Windows (SPSS, Inc., Chicago, IL, USA).

## Results

### Patient characteristics

A total of 336 critically ill patients admitted to the medical ICU between January 2006 and December 2009 were enrolled in the present study. Table 1 lists the reasons for ICU admission. The demographic data, clinical characteristics, and outcomes of the 2 score-matched groups are depicted in Tables 2 and 3, respectively.

**Table 1:** **Reasons for ICU admission**

	APACHE III-matched	SOFA-matched
	group (n = 174)	group (n = 110)
Sepsis	99(56.9%)	62(56.4%)
Urinary tract infection	9(5.2%)	8(7.3%)
Pneumonia	62(35.6%)	38(34.5%)
Intra-abdominal infection	16(9.2%)	7(6.4%)
Blood stream infection	9(5.2%)	8(7.3%)
Soft tissue infection	3(1.7%)	1(0.9%)
Cardiovascular diseases	4(2.3%)	2(1.8%)
Upper gastrointestinal bleeding	18(10.3%)	12(10.9%)
Hepatic failure	34(19.5%)	23(20.9%)
Other	19(10.9%)	11(10.0%)

*Abbreviation*: ICU, intensive care unit; APACHE, Acute Physiology and Chronic Health Evaluation; SOFA, sequential organ failure assessment.

**Table 2:** **APACHE III-matched patient demographic data and clinical characteristics according to cirrhosis and non-cirrhosis**

	Total (n = 174)	Cirrhosis (n = 87)	Non-cirrhosis (n = 87)	*p*-value
Age (years)	65.5 ± 1.1	59.8 ± 1.5	71.3 ± 1.4	<0.001
Male/Female	123/51	64/23	59/28	NS(0.405)
Length of ICU stay (days)	14 ± 1	11 ± 1	16 ± 2	0.009
Length of Hospital stay (days)	32 ± 2	30 ± 3	35 ± 3	NS(0.223)
Body weight on ICU admission (kg)	61 ± 1.0	65 ± 1	57 ± 1	<0.001
GCS, ICU first day (points)	9 ± 0	10 ± 1	9 ± 1	NS(0.077)
MAP, ICU admission (mmHg)	78 ± 1	80 ± 2	76 ± 2	NS(0.137)
Serum Creatinine, ICU first day (mg/dl)	2.5 ± 0.2	2.4 ± 0.2	2.5 ± 0.3	NS(0.885)
Arterial HCO_3_^−^, ICU first day	21 ± 1	19 ± 1.0	22 ± 1.0	0.002
Serum Sodium, ICU first day (mg/dl)	138 ± 1.0	138 ± 1.0	138 ± 1.0	NS(0.890)
Bilirubin, ICU first day (mg/dl)	6.4 ± 0.7	11.4 ± 1.3	1.4 ± 0.3	<0.001
Albumin, ICU first day (g/l)	2.4 ± 0.1	2.4 ± 0.1	2.4 ± 0.1	NS(0.329)
Blood Sugar, ICU first day (mg/dl)	166 ± 7	159 ± 11	170 ± 10.0	NS(0.482)
Hemoglobin, ICU first day (g/dl)	9.6 ± 0.2	9.2 ± 0.2	10.0 ± 0.2	0.024
Platelets, ICU first day (×10^3^/μL)	145.0 ± 9.1	79.4 ± 5.9	210.5 ± 14.1	<0.001
Leukocytes, ICU first day (×10^3^/μL)	14.5 ± 0.7	13.5 ± 1.0	15.5 ± 0.8	NS(0.133)
PaO_2_/FiO_2_, ICU first day(mmHg)	268 ± 11	275 ± 13	262 ± 17	NS(0.536)
Shock(%)	54 (31.0)	21 (24.1)	33 (37.9)	0.049
Hospital mortality (%)	114 (65.5)	64 (73.6)	50 (57.5)	0.026
*Score systems*				
APACHE III, ICU first day(mean ± SE)	87.8 ± 2.1	87.7 ± 3.4	88.0 ± 2.5	NS(0.941)
SOFA, ICU first day(mean ± SE)	9.6 ± 0.3	11.3 ± 0.4	8.0 ± 0.3	<0.001
RIFLE, ICU first day(mean ± SE)	1.6 ± 0.1	1.6 ± 0.1	1.7 ± 0.2	NS(0.913)

*Abbreviation*: NS, not significant; ICU, intensive care unit; SE, standard error; GCS, Glasgow coma scale; MAP, mean arterial pressure; PaO2, arterial partial pressure of oxygen; FiO2, fraction of inspired oxygen; APACHE, Acute Physiology and Chronic Health Evaluation; SOFA, sequential organ failure assessment; RIFLE, risk of renal failure, injury to the kidney, failure of kidney function, loss of kidney function, and end-stage renal failure.

**Table 3:** **SOFA-matched patient demographic data and clinical characteristics according to cirrhosis and non-cirrhosis**

	Total (n = 110)	Cirrhosis (n = 55)	Non-cirrhosis (n = 55)	*p*-value
Age (years)	65.0 ± 1.4	61.1 ± 2.0	68.9 ± 2.0	0.006
Male/Female	79/31	40/15	39/16	NS(0.832)
Length of ICU stay (days)	14 ± 1	11 ± 1	18 ± 2	0.007
Length of Hospital stay (days)	31 ± 2	32 ± 4	31 ± 3	NS(0.843)
Body weight on ICU admission (kg)	60 ± 1	64 ± 2	56 ± 2	0.001
GCS, ICU first day (points)	10 ± 0	11 ± 1	8 ± 1	0.005
MAP, ICU admission (mmHg)	78 ± 2	83 ± 2	73 ± 2	0.002
Serum Creatinine, ICU first day (mg/dl)	2.4 ± 0.2	1.8 ± 0.2	3.0 ± 0.3	0.004
Arterial HCO_3_^−^, ICU first day	21 ± 1	20 ± 1	21 ± 1	NS(0.458)
Serum Sodium, ICU first day (mg/dl)	137 ± 1	138 ± 1	137 ± 1	NS(0.282)
Bilirubin, ICU first day (mg/dl)	5.8 ± 0.8	9.6 ± 1.5	1.9 ± 0.4	<0.001
Albumin, ICU first day (g/l)	2.4 ± 0.1	2.5 ± 0.1	2.3 ± 0.1	0.016
Blood Sugar, ICU first day (mg/dl)	160 ± 8	159 ± 12	161 ± 11	NS(0.899)
Hemoglobin, ICU first day (g/dl)	9.7 ± 0.2	9.3 ± 0.3	10.0 ± 0.3	NS(0.134)
Platelets, ICU first day (×10^3^/μL)	127.3 ± 10.1	85.8 ± 8.1	168.8 ± 16.9	<0.001
Leukocytes, ICU first day (×10^3^/μL)	13.9 ± 0.8	12.9 ± 1.3	14.9 ± 1.0	NS(0.224)
PaO_2_/FiO_2_, ICU first day(mmHg)	256 ± 13	270 ± 16	243 ± 19	NS(0.299)
Shock(%)	39 (35.5)	9 (16.3)	30 (54.5)	<0.001
Hospital mortality (%)	71(64.5)	34 (61.8)	37 (67.3)	NS(0.550)
*Score systems*				
APACHE III, ICU first day(mean ± SE)	86.5 ± 2.8	81.1 ± 4.2	91.9 ± 3.7	NS(0.058)
SOFA, ICU first day(mean ± SE)	10.0 ± 0.3	10.0 ± 0.5	10.0 ± 0.3	NS(0.927)
RIFLE, ICU first day(mean ± SE)	1.6 ± 0.1	1.4 ± 0.2	1.9 ± 0.2	0.041

*Abbreviation*: NS, not significant; ICU, intensive care unit; SE, standard error; GCS, Glasgow coma scale; MAP, mean arterial pressure; PaO2, arterial partial pressure of oxygen; FiO2, fraction of inspired oxygen; APACHE, Acute Physiology and Chronic Health Evaluation; SOFA, sequential organ failure assessment; RIFLE, risk of renal failure, injury to the kidney, failure of kidney function, loss of kidney function, and end-stage renal failure.

In the APACHE III-matched group (Table 2), the critically ill patients with cirrhosis had a lower arterial HCO_3_^−^ level (*P* = .002), a lower haemoglobin level (*P* = .024), a lower platelet count (*P* < .001), and a higher serum bilirubin level (*P* < .001) on Day 1 of ICU admission compared with the patients without cirrhosis. The patients without cirrhosis with the same score were older and experienced more shock episodes than the patients with cirrhosis did. The hospital mortality rate was significantly higher in the patients with cirrhosis than in the patients without cirrhosis (*P* = .026). Renal function and a PaO_2_/FiO_2_ ratio were similar between the 2 groups. Among APACHE III-matched patients, the patients with cirrhosis had significantly higher SOFA scores than those of the patients without cirrhosis (*P* < .001).

In the SOFA-matched group (Table 3), the patients with cirrhosis had a lower platelet count (*P* < .001), a higher Glasgow coma scale (GCS), a more stable haemodynamic status, more favourable renal function, a higher bilirubin level, and a lower albumin level compared with the patients without cirrhosis. The patients without cirrhosis experienced more shock episodes than did the patients with cirrhosis. No significant difference in the hospital mortality rate was observed between the patients with and without cirrhosis.

In both the APACHE III and SOFA-matched groups, the patients with cirrhosis had a shorter overall length of stay in an ICU than that of the patients without cirrhosis. This was attributed to the higher hospital mortality rate in the patients with cirrhosis. In both the APACHE III and SOFA-matched groups, the patients without cirrhosis had a significantly higher rate of shock than the patients with cirrhosis did. This was because of the main reason that ICU admission of the patients with cirrhosis was mainly due to hepatic failure; the reasons for ICU admission of the patients without cirrhosis were GI bleeding and sepsis. (Data not shown here)

### Mortality and severity of illness scoring systems

Prediction abilities of the APACHE III, SOFA, and RIFLE scoring systems were compared; Table 4 lists the calibration and discrimination of the models. In the APACHE III-matched group, the SOFA scoring system demonstrated the highest prediction ability (AUROC = 0.810 ± 0.056) among all 3 systems. All the 3 scoring systems predicted mortality more precisely in the patients with cirrhosis than in those without cirrhosis. In the SOFA-matched group, the APACHE III scoring system was the most accurate predictor among all 3 systems. In the patients with cirrhosis, the SOFA scoring system demonstrated the highest prediction ability. To determine the selected cut-off points for predicting in-hospital mortality, the sensitivity, specificity, and overall accuracy of prediction were determined (Table 5). In the APACHE III-matched group, the SOFA scoring system had the best Youden’s index among the patients with cirrhosis, whereas the APACHE III scoring system had the best Youden’s index among the total population. In the SOFA-matched group, the APACHE III scoring system had the best Youden’s index among both the patients with cirrhosis and the total population. The RIFLE scoring system demonstrated the highest specificity for prognostic prediction in the patients with cirrhosis of both the SOFA-matched and APACHE III-matched groups.

**Table 4:** **Calibration and discrimination for the scoring methods in predicting hospital mortality**

	Calibration			Discrimination		
	goodness-of-fit (χ ^2^)	df	*p*	AUROC ± SE	95% CI	*p*
***APACHE III-matched group***						
*APACHE III*						
Total population	3.633	8	0.889	0.745 ± 0.040	0.667 – 0.823	<0.001
Cirrhosis	12.304	7	0.091	0.783 ± 0.062	0.662 – 0.904	<0.001
Non-cirrhosis	3.819	8	0.873	0.733 ± 0.055	0.626 – 0.840	<0.001
*SOFA*						
Total population	4.505	8	0.809	0.735 ± 0.039	0.659 – 0.812	<0.001
Cirrhosis	11.243	8	0.188	0.810 ± 0.056	0.700 – 0.920	<0.001
Non-cirrhosis	1.180	6	0.978	0.624 ± 0.060	0.506 – 0.741	NS(0.050)
*RIFLE*						
Total population	3.160	3	0.368	0.621 ± 0.045	0.534 – 0.709	0.010
Cirrhosis	2.008	2	0.366	0.710 ± 0.061	0.590 – 0.830	0.004
Non-cirrhosis	2.475	3	0.480	0.554 ± 0.062	0.431 – 0.676	NS(0.395)
***SOFA-matched group***						
*APACHE III*						
Total population	5.093	8	0.748	0.733 ± 0.051	0.633 – 0.834	<0.001
Cirrhosis	15.201	7	0.034	0.767 ± 0.068	0.633 – 0.900	0.001
Non-cirrhosis	1.716	7	0.974	0.706 ± 0.077	0.556 – 0.856	0.014
*SOFA*						
Total population	6.519	6	0.368	0.663 ± 0.053	0.559 – 0.767	0.005
Cirrhosis	6.427	6	0.377	0.742 ± 0.068	0.608 – 0.875	0.003
Non-cirrhosis	5.698	5	0.337	0.543 ± 0.079	0.387 – 0.698	NS(0.609)
*RIFLE*						
Total population	4.609	3	0.203	0.634 ± 0.055	0.527 – 0.742	0.020
Cirrhosis	1.216	2	0.544	0.656 ± 0.074	0.511 – 0.801	NS(0.053)
Non-cirrhosis	4.531	3	0.210	0.594 ± 0.083	0.432 – 0.756	NS(0.262)

*Abbreviation*: df, degree of freedom; AUROC, areas under the receiver operating characteristic curve; SE, standard error; CI, confidence intervals; APACHE, Acute Physiology and Chronic Health Evaluation; SOFA, sequential organ failure assessment; RIFLE, risk of renal failure, injury to the kidney, failure of kidney function, loss of kidney function, and end-stage renal failure.

**Table 5:** **Subsequent hospital mortality predicted after ICU admission**

Predictive Factors	Cutoff Point	Youden index	Sensitivity (%)	Specificity (%)	Overall correctness (%)
***APACHE III-matched group***					
*APACHE III*					
Total population	83	0.42	75	67	71
Cirrhosis	72^a^	0.55	78	76	77
Non-cirrhosis	83	0.40	70	70	70
*SOFA*					
Total population	10	0.40	52	88	70
Cirrhosis	10^a^	0.56	75	81	78
Non-cirrhosis	9	0.20	36	84	60
*RIFLE*					
Total population	Injury	0.22	39	83	61
Cirrhosis	injury^a^	0.35	45	90	68
Non-cirrhosis	Injury	0.10	32	78	55
***SOFA-matched group***					
*APACHE III*					
Total population	76	0.41	75	67	071
Cirrhosis	71^a^	0.50	74	76	75
Non-cirrhosis	83	0.40	73	67	70
*SOFA*					
Total population	10	0.30	45	85	65
Cirrhosis	10 ^a^	0.42	56	86	71
Non-cirrhosis	10	0.18	35	83	59
*RIFLE*					
Total population	Non-AKI	0.22	76	46	61
Cirrhosis	Injury^a^	0.26	35	90	63
Non-cirrhosis	Non-AKI	0.20	86	33	60

*Abbreviation*: APACHE, Acute Physiology and Chronic Health Evaluation; SOFA, sequential organ failure assessment; RIFLE, risk of renal failure, injury to the kidney, failure of kidney function, loss of kidney function, and end-stage renal failure.

^a^Value giving the best Youden index.

**Figure 1:**
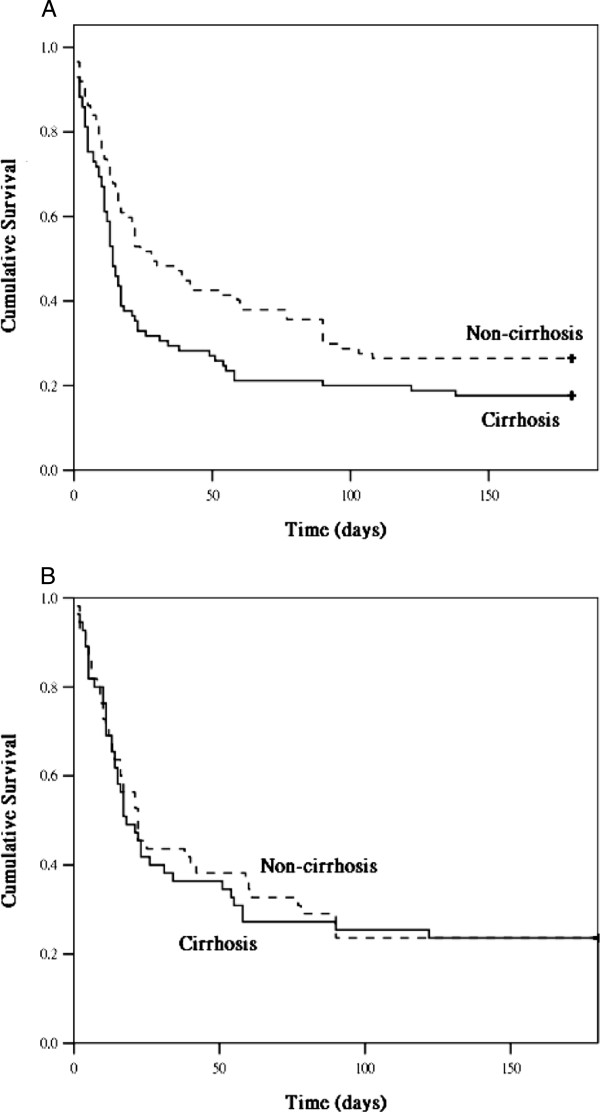
**The cumulative survival rates for cirrhotic and non-cirrhotic patients in the APACHE III-matched (1A, 174 patients,** ***p*** **< 0.05) and SOFA-matched (1B, 110 patients,** ***p*** **-value: not significant) groups, respectively.**

**Figure 2:**
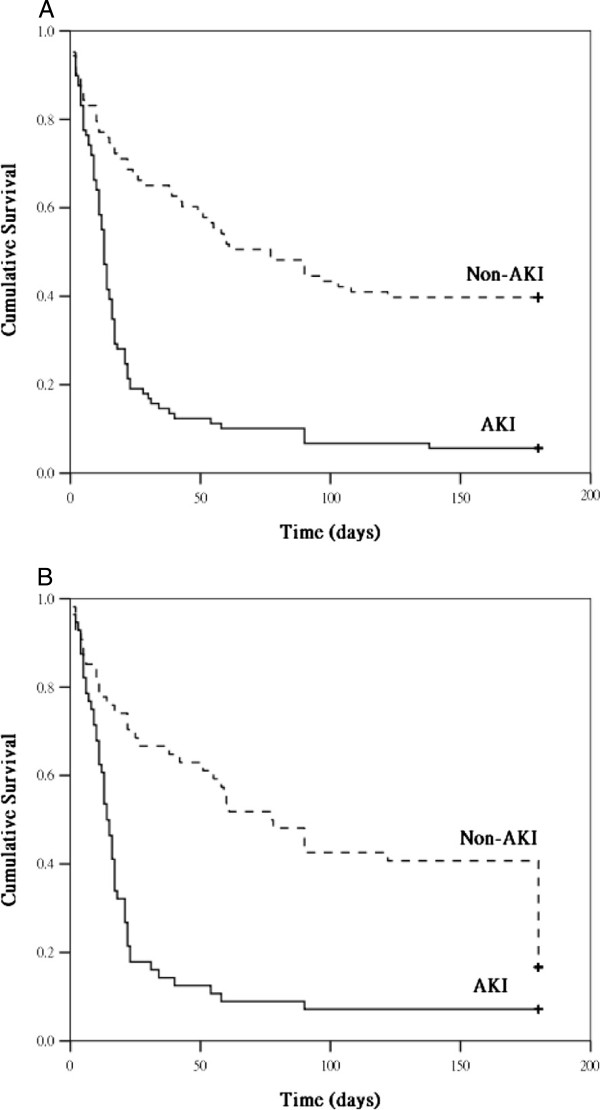
**The cumulative survival rates for acute kidney injury (AKI) and non-AKI patients in the APACHE III-matched (2A, 174 patients,** ***p*** **< 0.05) and SOFA-matched (2B, 110 patients,** ***p*** **< 0.05) groups, respectively.**

Figure 1A and B show the cumulative survival rates in the patients with and without cirrhosis in the APACHE III-matched and SOFA-matched groups, respectively. The cumulative survival rates showed that patients with cirrhosis in the APACHE III-matched group had significantly higher mortality rates than the patients without cirrhosis did, whereas no significant difference was detected between the patients with and without cirrhosis in the SOFA-matched group. In both the APACHE III-matched and SOFA-matched groups, the cumulative survival rates significantly differed when an underlying AKI was considered (Figure 2A and B).

## Discussion

Based on our research, this is the first study to compare the usefulness of different scoring systems for outcome prediction in patients admitted to an ICU with and without cirrhosis by using a score-matched method. In the present study, several clinical characteristics and outcomes of critically ill patients with cirrhosis were compared with those of critically ill patients without cirrhosis with matched APACHE III or SOFA scores. The poor outcome of patients with cirrhosis was consistent with the results of previous studies [12–14]. Recent studies have supported the efficacy of the SOFA scoring system for assessing the extent of organ dysfunction in various groups including critically ill patients with cirrhosis [3–5].

The APACHE III score consisted of the acute physiology score, age, and chronic health problem scores, which are widely used for predicting clinical outcomes currently. Among the patients with cirrhosis, bilirubin levels and incidences of cirrhosis were higher than those among the patients without cirrhosis. Results of the present study suggested that among patients with the same APACHE III score, patients with cirrhosis were older with a higher risk of acidaemia and shock than those without cirrhosis. However, renal function and consciousness status were nonsignificantly different between the 2 groups. The requirement for mechanical ventilation was not included in the APACHE III score, and no difference in the PaO_2_/FiO_2_ ratio was observed.

Although the SOFA score includes a fewer number of items and does not assess age and comorbid conditions, it enhances its simplicity and demonstrates high discriminatory power for predicting critically ill patients with cirrhosis. The patients with cirrhosis in the SOFA-matched group showed higher GCS and lower SCr concentrations on Day 1 of ICU admission, whereas these differences were nonsignificant in the APACHE III-matched group. Because patients with cirrhosis typically have higher serum bilirubin levels and lower platelet counts (caused by de novo liver disease), which contribute to a higher SOFA score, than their counterparts do, the patients with cirrhosis with similar SOFA scores may have relatively improved characteristics in the other 5 organic fields. This phenomenon is consistent with our findings showing that the patients with cirrhosis had a more stable neurologic status and renal function than did the patients without cirrhosis in the SOFA-matched group.

APACHE III, a prognostic model, predicts mortality. Prognostic scoring models such as APACHE III assume that mortality is affected by physiological disturbances that occur early in the course of illness, whereas organ dysfunction-scoring systems such as SOFA allow determination of organ dysfunction at the time of admission and at regular intervals throughout the stay in an ICU, thus allowing for the assessment of changes in organ function. The SOFA score is an organ dysfunction score that quantifies the burden of organ dysfunction. Although the SOFA score was originally used to describe morbidity, it was also used in mortality prediction. The accuracy of mortality predictions may be improved with repeated measurements by using organ dysfunction scoring systems such as SOFA.

As shown in Table 4, in the APACHE III-matched group, the SOFA score demonstrated a higher discrimination ability in the patients with cirrhosis than in the patients without cirrhosis (AUROC = 0.810 ± 0.056 vs 0.624 ± 0.060). The SOFA score is simpler for assessment than the APACHE III score by clinicians. Meanwhile, the SOFA score allows for sequential measurements and more accurately reflects the dynamic aspects of disease processes and may provide information of higher quality on the mortality risk. Therefore, the SOFA score is a superior and easier-to-implement model for predicting mortality in the patients with cirrhosis, with a cut-off value of 10 points, providing the optimal overall correctness. In addition, both the SOFA and APACHE III scores are comparable in the patients without cirrhosis.

When the patients with and without cirrhosis were matched by using SOFA scores, difference in APACHE III scores between the 2 groups was nonsignificant in the present study. The RIFLE scoring system, however, showed superior results in patients with cirrhosis to those of the noncirrhotic controls (*P* = .041) in the SOFA-matched group. The patients with cirrhosis tend to have malnutrition, low muscle mass, and impaired synthesis of creatinine. Therefore, the RIFLE scoring system, which is based on the serum creatinine and urine output, may lead to underestimation of AKI severity and overall illness.

Despite the encouraging results of the present study, several potential limitations should be considered. First, this was a retrospective study performed at a single tertiary-care medical centre, which limits generalisation of the findings. Second, the patients without cirrhosis mainly composed of patients with sepsis and those with a low proportion of patients with other diseases such as cardiovascular disease or acute respiratory distress syndrome. The specificity of the subgroup of the patients with cirrhosis may limit the generalisation. Third, the patient population comprised a high proportion of patients with hepatitis B virus infection. Therefore, this study has limited applicability to typical North American and European patients with hepatitis C virus infection or those with alcohol dependence. Finally, the sample size was insufficient for matching SOFA and APACHE III scores among the patients with and without cirrhosis. Therefore, we cannot draw definitive conclusions regarding the relatively poor short-term prognosis of the patients admitted to the ICU with cirrhosis compared with that of the patients admitted to the ICU without cirrhosis.

## Conclusions

Our results provide additional evidence that SOFA scores differ significantly between patients with and without cirrhosis matched according to APACHE III scores. The score-matched analytical data showed that the predictive accuracy of SOFA is superior to that of APACHE III in evaluating critically ill patients with cirrhosis. We also demonstrated that the mean arterial pressure, GCS, and RIFLE classification play critical roles in determining prognosis in this subset of patients. When considering cost-effectiveness and ease of implementation, the SOFA scale is recommended for evaluating short-term prognosis in critically ill patients with cirrhosis.

## Abbreviations

ICU: intensive care unit
APACHE III: Acute Physiology and Chronic Health Evaluation III
SOFA: Sequential Organ Failure Assessment
RIFLE: Risk of renal failure, Injury to the kidney, Failure of kidney function, Loss of kidney function, and End-stage renal failure
AKI: acute kidney injury
APS: acute physiology score.
